# Mixed-method study of a conceptual model of evidence-based intervention sustainment across multiple public-sector service settings

**DOI:** 10.1186/s13012-014-0183-z

**Published:** 2014-12-10

**Authors:** Gregory A Aarons, Amy E Green, Cathleen E Willging, Mark G Ehrhart, Scott C Roesch, Debra B Hecht, Mark J Chaffin

**Affiliations:** Department of Psychiatry, University of California, La Jolla, San Diego, CA USA; Pacific Institute for Research and Evaluation, Albuquerque, NM USA; Department of Psychology, San Diego State University, San Diego, CA USA; Department of Pediatrics, University of Oklahoma Health Sciences Center, Oklahoma City, OK USA; School of Public Health, Georgia State University, Atlanta, GA USA

**Keywords:** Sustainment, Evidence-based intervention, Implementation, Leadership, Organizational culture, Organizational climate, Policy, Public sector

## Abstract

**Background:**

This study examines sustainment of an EBI implemented in 11 United States service systems across two states, and delivered in 87 counties. The aims are to 1) determine the impact of state and county policies and contracting on EBI provision and sustainment; 2) investigate the role of public, private, and academic relationships and collaboration in long-term EBI sustainment; 3) assess organizational and provider factors that affect EBI reach/penetration, fidelity, and organizational sustainment climate; and 4) integrate findings through a collaborative process involving the investigative team, consultants, and system and community-based organization (CBO) stakeholders in order to further develop and refine a conceptual model of sustainment to guide future research and provide a resource for service systems to prepare for sustainment as the ultimate goal of the implementation process.

**Methods:**

A mixed-method prospective and retrospective design will be used. Semi-structured individual and group interviews will be used to collect information regarding influences on EBI sustainment including policies, attitudes, and practices; organizational factors and external policies affecting model implementation; involvement of or collaboration with other stakeholders; and outer- and inner-contextual supports that facilitate ongoing EBI sustainment. Document review (e.g., legislation, executive orders, regulations, monitoring data, annual reports, agendas and meeting minutes) will be used to examine the roles of state, county, and local policies in EBI sustainment. Quantitative measures will be collected via administrative data and web surveys to assess EBI reach/penetration, staff turnover, EBI model fidelity, organizational culture and climate, work attitudes, implementation leadership, sustainment climate, attitudes toward EBIs, program sustainment, and level of institutionalization. Hierarchical linear modeling will be used for quantitative analyses. Qualitative analyses will be tailored to each of the qualitative methods (e.g., document review, interviews). Qualitative and quantitative approaches will be integrated through an inclusive process that values stakeholder perspectives.

**Discussion:**

The study of sustainment is critical to capitalizing on and benefiting from the time and fiscal investments in EBI implementation. Sustainment is also critical to realizing broad public health impact of EBI implementation. The present study takes a comprehensive mixed-method approach to understanding sustainment and refining a conceptual model of sustainment.

**Electronic supplementary material:**

The online version of this article (doi:10.1186/s13012-014-0183-z) contains supplementary material, which is available to authorized users.

## Background

Evidence-based interventions (EBIs) are increasingly being implemented in public-sector health and allied health service settings with little systemic knowledge about what factors facilitate or limit their sustainment. Without effective sustainment, investments in implementation are wasted and public health impact is limited. In the United States, the National Institutes of Health, Agency for Healthcare Research and Quality, Centers for Disease Control and Prevention (CDC), and other federal and state agencies and foundations are funding studies to facilitate more effective implementation of EBIs, but there are few systematic studies of sustainment. Adding to the need for research on sustainment is the fact that many EBIs are not sustained after initial implementation [1]-[4]. Leadership, policies, resource availability, collaborations, and organizational infrastructure are proposed as key determinants of long-term sustainment, yet these elements have not been widely examined [5],[6]. This current study is consistent with conceptual models that propose multiple phases or stages in the implementation process [5],[7]-[9], and focuses explicitly on the period of sustainment. Prospective and retrospective mixed methods are combined to examine three broad issues believed to be critical to EBI sustainment: 1) policies at the legislative, service system, and agency levels; 2) collaborations and partnerships; and 3) long-term bi-directional organizational and individual provider predictors of sustainment outcomes of reach/penetration, fidelity, and sustainment climate [5],[10].

Although some models of implementation invoke sustainment as a key component [1], little empirical work has systematically examined factors that either facilitate or hinder EBI sustainment in public-sector services [11]. In a comprehensive review of the sustainment literature, both outer (system) and inner (organizational) contexts emerged as dominant features across conceptual models [12]. In particular, Klein and colleagues [13] identified management support, organizational supports, and fiscal resources as key elements for implementation effectiveness and sustainment. Additionally, Edmondson [2] found leadership and positive team climate to be key factors in sustainment. Pertinent to the outer context, Pluye and colleagues [14] emphasized policies and processes as precursors to sustainment. Finally, Chambers and colleagues [10] proposed a model of sustainment that spans the ecological system (e.g., systems and policies) and practice setting (e.g., organizational context, service providers).

**Figure 1:**
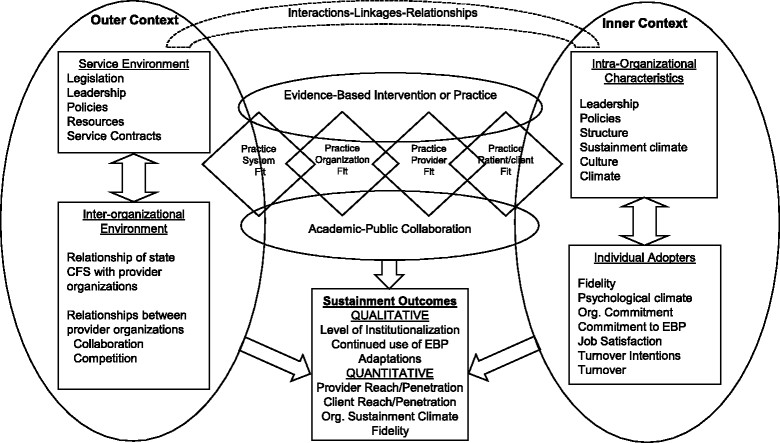
**Sustainment conceptual model based on the Exploration, Preparation, Implementation, Sustainment (EPIS) multilevel conceptual framework (Aarons, Hurlburt, & Horwitz, [**[11]**]).** Note: some constructs may be considered predictors or outcomes dependent on particular hypotheses or research questions.

For this study, we build on the Exploration, Preparation, Implementation, Sustainment (EPIS) implementation framework [5] to guide our conceptualization of factors in both outer (i.e., system) and inner (i.e., organization) context factors that affect sustainment. As shown in Figure 1, our proposed conceptual model of sustainment illustrates factors in the outer system and inner organizational contexts that interact and involve relationships among multiple stakeholders including federal and state governments and community-based organizations (CBOs, also known as non-governmental organizations or NGOs), in addition to collaborations with other community stakeholders, academic researchers including EBI developers and purveyors, and funding agencies that support the reach of the EBI to providers and client populations and achieve EBI model instantiation in service systems and, ultimately, EBI model fidelity [5].

### Outer-context issues in EBI sustainment

Conceptual models and reviews identify common outer-context (i.e., system) factors that can influence the capacity of systems and organizations to successfully implement and sustain EBIs [5],[11],[15],[16]. Governmental actions are particularly salient for EBI implementation and sustainment [17]. As such, the outer context of our conceptual model focuses on legislation, policies, public-sector fiscal resource availability, bid solicitations, reimbursement schemes, and how these factors are instantiated into service contracts. Legislation affects services by mandating funding streams or types of services to be provided [18]. Policies within state and county divisions allocate funding and set the parameters for service delivery (e.g., mandating EBIs) [19]. Service contracts can be structured to promote or reflect priorities of the state agency and represent an important mechanism for influencing organizational behaviors regarding EBIs [20]. Bid requirements can specify particular EBIs or leave this to CBO discretion. Reimbursement schemes can support key EBI activities such as quality control or fidelity monitoring to various degrees. Contractual arrangements are common in public-sector services as state or county governments rely on CBOs to provide services beyond their scope or expertise [5]. Such a multifaceted approach is likely necessary to sustain EBIs on a large scale [19].

### Inner-context issues in EBI sustainment

Organizational and individual (i.e., provider) factors are important when implementing EBIs in real-world service contexts, and it is likely that these are at play in sustainment as well [21],[22]. For example, the social influence of others in an organization or work team can impact worker attitudes toward practice models [23],[24], and attitudes can affect implementation outcomes such as adoption and use of EBIs [25],[26]. The level of tangible organizational support for a practice is likely to affect adoption, implementation, and sustainment of EBI [13],[22]. The culture and climate of an organization or work team are associated with service provider engagement in work and willingness to utilize EBIs [27],[28], as are individual provider characteristics, including job tenure and level of professional development [29] and attitudes [25]. Strategic organizational climates can support specific employee behaviors [30]-[32], such as EBI use and fidelity. Leadership that motivates providers and promotes effective interaction is linked to service provider attitudes toward adopting EBI [33]. Professional and social networks within service systems spread knowledge about the perceived utility of an EBI and are likely to impact the quality of EBI uptake [34]. A service provider’s own ability to adapt and change can shape attitudes toward adopting new ways of working among his/her colleagues [29]. Finally, it is important to examine measures of strategic climates focused on implementation and sustainment. Sustainment climate is characterized by strong organizational practices, processes, and leader support for EBI that demonstrate what is expected, supported, and rewarded in an organization [32],[35].

### Collaboration/partnerships

Linkages between systems, organizations, their environments, and resources (e.g., financial, workforce, and knowledge) are needed for EBI implementation and sustainment [11]. Partnerships involving service system stakeholders can facilitate linkages during the implementation process and promote both positive EBI outcomes [16] and subsequent intervention sustainment [36]. In the context of implementing EBIs through multiple public-private service contracts with CBOs, the involvement of academic partners with a history of community and public-sector engagement can foster strong and supportive relationships among institutions and individual professionals. Although resulting partnerships are not always the product of a planned community-based participatory research [37],[38] or community-partnered participatory research initiative [39], they nonetheless appear to help align the interests of the policymakers, upper-level CBO administrators, direct service providers, and academic researchers in order to undertake activities that promote both initial and ongoing fit of the selected EBI within a service system in the face of competing interests and priorities. The World Health Organization recommends such partnerships as a viable means to implement and sustain services [40]. Interest in such partnerships for intervention development and implementation purposes is also blossoming within mental health services research [41]-[43].

### The EBI

The current study contributes to implementation science by addressing the sustainment of SafeCare© (SC), a home-based, behavioral and psychosocial EBI developed to prevent child neglect, a pressing public health problem and by far the most common type of child maltreatment [44]. Child neglect results in negative health, emotional and behavioral effects, and a large economic burden on society [45]. Neglected youth have a higher likelihood of mental health problems including social and emotional withdrawal [46],[47], low self-esteem, and less confidence and assertiveness in learning tasks [48]. They also present the least positive and the most negative affect of all maltreated children [48]. Consequences of neglect include poor academic performance, risk for developmental delays and cognitive difficulties [49], delayed language development [50],[51], and delays in receptive and expressive language [52]. Followed into adulthood, neglected children are at high risk for substance abuse and related behaviors, such as violence and criminal behavior [53]. In addition, while physical and sexual abuse rates have declined markedly over the past 18 years, child neglect rates have remained high with 32.6% of child maltreatment deaths attributable to neglect only [54]. Thus, addressing neglect is a critical public health concern affecting health, mental health, and child development and subsequent adult functioning.

The SC model incorporates principles of applied behavior analysis, is manualized, and uses classic behavioral intervention techniques (e.g.*,* ongoing measurement of observable behaviors, skill modeling, direct skill practice with feedback, training skills to criterion) [55]. Behavioral theory conceptualizes child neglect in terms of caregiver skill deficits, particularly those skills that are most proximal to neglect, such as failing to provide adequate nutrition, healthcare, cleanliness, a safe home environment, parental disengagement, low levels of parental supervision, and inappropriate parenting or child management. SC is comprised of three modules addressing these issues: infant and child health, home safety and cleanliness, and parent-child (or parent-infant) interactions and skills development in problem solving and communications. The EBI has been found to reduce child welfare system recidivism, over and above comparable services without the SC curriculum when implemented at scale [44], making sustainment of this benefit a significant public health imperative. SC involves training providers and having ongoing fidelity coaching from SC model experts. Finally, the United States National SafeCare Training and Research Center (NSTRC) has developed a “train-the-trainer” model, in which selected providers can be trained and certified as SC coaches and trainers to expand or sustain local implementation [56].

### The need for studies of sustainment

The study of sustainment is critical. For example, a review of home-based services found a 55% failure rate for implemented programs (i.e., services completely stopped being provided). Of those programs that were still “identifiable”, many of the key treatment elements (i.e., core elements) were no longer part of the services [57]. This highlights the need to increase our knowledge of how to effectively sustain EBIs with fidelity. Sustainment of EBIs to decrease neglect is critical for decreasing a child’s risk for neglect and resulting mental health, substance abuse, mortality, and decrements in health functioning.

The study of sustainment is not a well-developed science; however, there is a small existing literature on sustainment of prevention and intervention programs in a variety of settings that is informative. For example, studies of sustainment of school-based programs have found that variability in implementation success was a function of the quality of the relationships of educators with program recipients [58], that academic-community partnerships to support implementation can be maintained over time [59], and that action-research approaches emphasizing stakeholder involvement in the research process can aid sustainment [60]. Research in community-based settings has found that sustainment is enhanced when costs are shifted from services as usual to the new service model without an overall increase in costs [61]. In addition, contextual factors at both the system and organizational levels can be important through diffusion stages [16], and system issues can exert a greater effect relative to individual provider factors [62]. Viewing implementation as a developmental process across EPIS stages, it is likely that different factors assert critical influences at each stage. There is a need to identify unique sustainment factors across system, organization, and individual levels that can lead to improvements in processes and efficiencies not considered during initial implementation [63]. Such factors should span outer and inner contexts and might include engaging strong leadership across system and organizational levels, use of specific management strategies, attending to both organizational and individual factors, and anchoring new programs across system levels [64]. It is also imperative to look across levels, because system level instability negatively impacts sustainment [65]. For example, workforce policies may need to be tailored differently for urban and rural settings [66], and alternative funding sources may be necessary in some systems for particular types of interventions [67].

### The present study

This study of EBI sustainment is funded by the U.S. National Institute of Mental Health (NIMH) and received institutional review board approval from the University of California, San Diego. The study builds on previous studies of implementation of SC [44],[68]-[70] in two U.S. states. Implementations began between 1 and 10 years prior to the beginning of the current study providing variability in sustainment duration. Additionally, implementations spanned multiple types of service systems including state/county departments of mental health, public health, and social services. This study examines sustainment from three complementary perspectives of policy, collaboration, and organizational and provider functioning. Further, this study utilizes mixed methods, involving stakeholders from the outer (i.e., service system) and inner (i.e., provider organizations) contexts, along with academic collaborators, to examine factors related to sustainment and refine a conceptual model of EBI sustainment.

## Method/design

### Study context

In this study, we examine sustainment of SC after implementation in 11 public-sector service systems across two states and 87 counties. One service system is state-operated with all services provided by CBOs contracted by the state government. The other ten have county-operated systems, in which services are guided, contracted, and/or delivered by county governments. The statewide implementation began 10 years prior to the inception of the current study with continuous involvement from university researchers as part of large-scale NIMH-funded effectiveness [44] and implementation [69] studies. The county-wide implementations were funded by the CDC, the NIMH, the Administration for Children and Families, state government, and community charitable foundations. These implementations involved university and community collaborations with specific projects to examine: 1) a cascading model of implementation featuring inter-agency collaborative teams [71],[72] and 2) utilization of the Dynamic Adaptation Process [73] to facilitate implementation. For the first study, EBI implementation began in a single large county 6 years prior to the onset of the current study. The second study involved ten counties that began implementation between 1 and 3 years prior to this study. Next, we present the specific aims and methods for accomplishing each aim.

### Specific aims

#### Aim 1

Examine the impact of state and county policies and contracting on the provision and sustainability of an EBI within the publicly-funded service systems.

##### Document review

Documents offer a rich source of information on the intended and actual role of state policy in influencing the uptake and sustainment of SC and the on-the-ground effects of policies within service systems and service organizations. Document collection and analysis procedures will be applied to assess the outer context of EBI sustainment, described in Figure 1[74],[75]. First, EBI-specific documents released by the state and county government between the start of SC implementation and the present, including legislation, executive orders, governor’s speeches, regulations, monitoring data, and annual reports, will be systematically collected and indexed. Agendas and minutes for all meetings related to SC, in addition to request for proposals (RFPs) and contracts with CBOs for SC provision, will also be collected. Over the course of this 5-year study, these documents will continue to be updated quarterly.

Traditional methods in both archival and qualitative research will be utilized to prepare and analyze the documents. Upon retrieval, each document will be entered into a computer database, assigned a unique identification number, and indexed according to type, purpose, or reason for creation/issuance, source, and date. An abstract that provides a brief description or summary of contents will be created for each document. Coding schemes will be developed by the research team for each type of document (e.g., legislation, legal action, etc.). This coding scheme will facilitate further categorization of document content. For each document type or group, two researchers will randomly select a sample of the various documents and generate a list of codes or content categories relevant to SC sustainment [76]. Document contents that do not appear immediately relevant to the research topic at hand will be placed in a separate category, or “parking lot”, for possible analysis in the future. This standard set of codes will then be applied to the broader group. For within-group analyses, changes in contents for specific documents (e.g., modified implementation requirements within CBO contracts and subcontracts) we be identified and analyzed. Analysis of documents will serve several purposes. For example, the review and comparison of enabling legislation and annual RFPs and contracts will help to retrospectively and prospectively trace how SC has matured over time. Analysis of other documents may lend insight into the partnership dynamics explored under Specific Aim 2. For example, iterative review of meeting minutes may shed light on those partners who are most active in the SC initiative, their roles and responsibilities, leadership and infrastructure issues, as well as planning and decision making processes [36]. Document review will facilitate understanding in-depth the relationship between official pronouncements concerning SC and its statewide implementation and factors that might affect reach/penetration and fidelity. Findings derived from the document review will also stimulate “paths of inquiry” to pursue via one-on-one and small group interviews with a broad array of SC stakeholders (see below) [77].

##### Semi-structured interviews

Open-ended questions will be used to collect descriptive data on the development of policies that have influenced widespread adoption and utilization of SC. The experiences, motivations, and perceptions of state/county administrators, academic investigators, CBO executive directors, and regional directors involved in implementing these policies will also be assessed. Interview questions will elicit information on the current positions and professional backgrounds of each participant; their respective roles in and shared vision for SC implementation, evaluation, and dissemination; the relative success of SC-related legislation, policies, and service contracts in facilitating these processes; and factors that will “make or break” sustainment of SC in the future. These approximately 1.5-h, digitally-recorded interviews will provide an opportunity for participants to contemplate local circumstances (e.g., availability and access to community-based resources) and non-SC contextual factors at the macro-level that might affect sustainment (e.g., yearly fluctuations in federal funding for programs, changes in state economies, changes in political administration, etc.). Handwritten field notes will be organized according to a standard format (“debriefing form”), which includes information on date, time, length of the interaction, physical setting, and participants involved [78],[79]. Field notes and interview transcripts will be converted into analyzable text and stored electronically in a password-protected computer database.

#### Aim 2

Investigate the role of public, private, and academic relationships and collaboration in the long-term sustainment of an EBI among multiple public-sector service systems.

Qualitative and quantitative methods will be used to assess the role of public, private, and academic relationships in facilitating sustainment of EBIs. Semi-structured interviews will be used with state/county administrators, academic investigators, CBO executive directors, and CBO directors to examine the nature, quality, and degree of formalization of collaborative relationships that have facilitated large-scale implementation of SC in the public sector, and how these relationships might affect the level of institutionalization and sustainment of this intervention in the service system [80]. Thus, this examination will focus on three levels: 1) infrastructure, functions, and processes of extant (formal and informal) partnerships; 2) accomplishment of SC-related activities undertaken by these partnerships; and 3) impact of these activities on EBI sustainment. As part of these interviews conducted over the course of the study, we will collect information on the history of naturally evolving or devolving collaborations that have affected SC sustainment, as well as factors that have positively or negatively affected these collaborations over time (e.g., trust issues, power differentials, and prior partnerships) [81]. Participants will be asked to reflect upon their own involvement in SC-related collaborations, how these collaborations developed over time, and outcomes or accomplishments made possible by these collaborations. Participants will be prompted to discuss the types of interactions they have had with each set of SC stakeholders and how these interactions have affected (or will affect) SC sustainment. Stakeholder participants will be asked about specific processes that are identified in the literature as pivotal to successful collaborations (e.g., communication, problem solving, decision making, consensus-building processes, and conflict management and resolution) and how these processes play out in SC partnerships [36]. In addition to collaboration, participants will comment on other factors likely to influence SC institutionalization and the sustainment climate, including leadership at multiple levels (state/county, academic, CBO, and SC team), evaluation, infrastructure, and funding.

#### Aim 3

Assess a targeted range of organizational and individual provider factors within direct service agencies that affect reach/penetration, fidelity of SC, and organizational sustainment climate over time.

##### Semi-structured interviews

SC coaches and CBO supervisors will participate in one-on-one 1-h interviews to document their overall perceptions and experiences with SC and views regarding long-term sustainment. Participants will be asked about their general involvement in SC (including history), probing specifically for positive and negative experiences and challenges encountered during the implementation process that impact SC sustainment. Participants will then be guided toward how the SC model is applied within CBO settings, changing attitudes regarding the model, and changes within CBOs that have influenced attitudes. Third, participants will be asked to provide more targeted commentary on organizational factors within CBOs that affect SC model sustainment on a day-to-day basis, including team functioning and leadership, funding, and external policies (e.g., contract requirements, state regulations, legal actions). Fourth, participants will address prior or current involvement with non-CBO SC stakeholders, including participation in formal or informal collaborations. Finally, participants will comment on the type of support and resources needed within CBOs and teams to effectively provide SC on an ongoing basis. These interviews will also identify “lessons learned” during the coaching and supervisory process, which could help guide future implementation to support EBI sustainment.

##### Group interviews

Group interviews will be conducted with service providers assigned to SC teams [77]. Group interviews allow for the inclusion of the voice of each participant and are efficient in that trained discussion facilitators will be on-site when teams regularly meet, thus reducing respondent and CBO burden. Each work group will be comprised of 4–10 providers. Each group interview will be conducted over a 1–2-h period with the facilitator guiding the participants in a process of collective reflection and evaluation of issues pertinent to the institutionalization of SC within the service system and CBOs. The questions posed to group participants will parallel those asked of the coaches and supervisors and will thus focus on general involvement, changing attitudes and practices regarding SC, organizational factors and external policies affecting model implementation, involvement or collaboration with other SC stakeholders, and outer- and inner-contextual support needed to facilitate ongoing implementation of SC on individual and organizational levels.

##### Qualitative data preparation

Both the individual and group interviews will be digitally recorded, transcribed, and coded according to the procedures described above for Aim 1; debriefing forms will be completed for each interview. Similarly, transcripts will be analyzed according to the previously described qualitative methods. We will cluster for analysis those responses about factors at the policy, partnership, and organizational level that are likely to affect sustainment of SC [77]. The researchers will then prepare a summary of issues, ideas, and concerns about sustainment raised by the providers, which will be addressed during the next phase of the research, the development of a conceptual model.

##### Qualitative data analysis

To analyze the interviews, a descriptive coding scheme will be developed from transcripts and based on the specific questions and broader domains that make up the interviews. NVivo software will be used to organize and index data and aid in the identification of emergent categories and themes [78],[79]. Two types of coding will be used. First, “open coding” will be used to locate themes followed by “focused coding” to determine which themes repeat often and which represent unusual or particular concerns [82]. Coding will proceed in an iterative fashion; we will code sets of transcripts, create detailed memos linking codes to emergent themes, and review with the project’s lead investigators. Discrepancies in coding and analysis will be identified during this review process and resolved during regular team meetings.

Throughout this project, the consistency of interview data collected at different times (Years 1, 3, and 5) and by different methods will be assessed. More specifically, the procedures will utilize a) cross-checking interview data collected by individual staff, b) comparing interview data with findings from the document review, c) checking for constancy in what participants say about the implementation and sustainability of SC over time, and d) comparing perspectives of different stakeholder groups, e.g., state/county, academic, CBO.

##### Triangulation of qualitative data sources

Triangulation of findings derived from document review with those from individual and group interviews will be enacted to create a complete picture of sustainment issues to date. Interviews will shed light on “behind-the-scenes” events and processes that led to the establishment and dissemination of SC policy and practice and the range of outer- and inner-contextual variables likely to influence further sustainment. The combination of qualitative methods enables us to answer several questions concerning SC sustainment: 1) How does county/state-level legislation impact the service system? 2) How does this system implement policies that result in the issuance of RFPs and service contracts with CBOs? 3) How do CBOs within the service system then collaborate and compete to provide EBIs, such as SC? 4) How do CBOs carry out the terms and conditions of contracts to provide SC? 5) What organizational characteristics within CBOs affect individual providers, team structure and operations, and delivery of SC? 6) What system, organizational, and EBI adaptations support sustainment?

### Quantitative methods

#### Participants and measures

Quantitative data will include administrative and service delivery data provided by the service system representatives, organizational and individual measures collected from direct service providers and supervisors using annual online surveys, and administrator and executive director key informant measures.

#### Measures from administrative data

Three measures from administrative data will be collected:

1. *Reach/penetration - provider level*. We will assess the proportion of providers who have a) received training in SC, b) reached certification, and c) have successfully completed SC services with clients (closed cases only).
2. *Reach/penetration - client level*. We will use de-identified administrative data to determine the annual number of potential cases meeting criteria for referral to SC, proportional to the number of cases actually receiving SC.
3. *Staff turnover*. Data will be gathered from administrative data and/or organization and provider reports. Post-turnover follow-up calls with SC providers will be used to gather more detail regarding reasons for turnover (e.g., system changes, voluntary or involuntary turnover, other job opportunities, family issues, concerns with the EBI).

#### Coach measures

Measures of SC model fidelity will be collected from SC coaches. Provider fidelity is a critical sustainment outcome [15] that impacts clinical outcomes [83]. Directly observed fidelity is most related to client outcomes because observers normally are fluent with the constructs and behaviors they are rating. SC coaches are model experts trained in effective coaching practice and SC fidelity ratings. Observations will be coded by coaches using a SC fidelity measure for each observed session (one to four sessions per provider monthly for a minimum of 12 observations per year per provider).

#### Provider measures

Data will be collected from providers using web-based surveys. The following measures will be included in the provider survey:

1. *Organizational culture, climate, and work attitudes* will be assessed with the Organizational Social Context Survey [84].
2. *Leadership* will be assessed with the Multifactor Leadership Questionnaire [85] and the Implementation Leadership Scale [86].
3. The *knowledge/perceived value of SC* will be assessed within the study workforce with 16 questions adapted from qualitative and quantitative studies examining service system functioning [34],[87],[88].
4. *Sustainment climate* is a strategic climate that captures the provider perceptions of the extent to which the policies, practices, and procedures in the organization support sustainment. It will be assessed using an adaptation of the Implementation Climate Scale [32] which is comprised of 18 items with six subscales and which has excellent psychometric characteristics (*α* for subscales range = .81–.91).
5. *Attitudes toward evidence-based practice* will be assessed with the Evidence-Based Practice Attitude Scale [29],[89],[90] (total *α* = .76).

#### Administrator measures

Data will be collected from administrators using web-based surveys. The following measures will be included in the administrator survey:

1. Five subscales from the *Program Sustainability Index* [91],[92] will assess *leadership competence* (*α* = .81, 5 items), *effective collaboration* (*α* = .88, 10 items), *program results* (*α* = .85, 4 items), *strategic funding* (*α* = .76, 3 items), and *staff involvement* (*α* = .76, 4 items).
2. The *Level of Institutionalization Scale* [80] will be adapted for this study to assess the degree to which SC is institutionalized in the service system and provider organizations using the *Cycles-Routines* (*α* = .87) and *Niche Saturation* scales (*α* = .84).

#### Quantitative analyses

All data analytic strategies will adhere to recommendations of the Prevention Science and Methodology Group [93]. Primary analyses will be based on generalized linear mixed models [94]-[96], because the data will have a three-level hierarchical data structure in which measurements over time are nested within individuals (i.e., providers), and individuals are nested within supervision groups. In general, the approach to modeling this type of multilevel data will consist of including random coefficients at the provider and supervision group levels. While this approach is typically sufficient to control for the dependency among observations, we will also test for the possible presence of residual dependency by fitting models that superimpose different autocorrelated error structures on the baseline random-effects models and compare the fit of these nested models using a likelihood ratio test [94],[96]. The models tested involve both fixed and time-varying covariates. Our modeling approach imposes a 1-year lag on observations for the response (e.g., for time-varying covariates, Time 1 values are used to predict Time 2 values on the response, Time 2 values on the covariate are used to predict time 3 values on the response, controlling for Time 1 measurements on the response) [97]. Given that the conventional mixed model imposes the untested constraint that each time-varying covariate has equal between- and within-subject effects, we will explore models that provide separate estimates of these effects and thereby relax this constraint [94],[98]. All models will be multivariate in nature (i.e., testing all predictors simultaneously). Significance tests will focus on individual regression coefficients from these models.

Provider reach/penetration is a binary indicator (either the provider is trained, certified, and is delivering every indicated module of SC to clients on their caseload or not) and will be modeled with a logit link function. Demographic variables (age, gender, race/ethnicity, education level, years of experience, job tenure, urban/rural), work attitudes, turnover intentions, team turnover rate, organizational climate, and sustainment climate will be included in the model. Given that the response and predictors are measured at each assessment period, we will lag the response, controlling for baseline measurements of the response [97].

Client reach/penetration will be evaluated with two statistical models. Client penetration is conceived as the number of clients being served by SC divided by those served by SC plus those who need SC, but are not served (i.e., served/[served + unmet need]). The proportion of clients served with SC in each implementation site will be compared using a chi-square goodness-of-fit test, followed by comparisons among the categories conditional on a significant overall test. These comparisons will be conducted cross-sectionally. There is no hierarchical structure associated with this outcome. In the presence of statistically significant differences between sites, qualitative analyses will be conducted to identify cause of the disparity.

Fidelity ratings of SC providers will be treated as a continuous outcome, modeled using a link function selected on the basis of the observed distribution. Predictors include work attitudes, organizational climate, sustainment climate, and demographics variables. While predictor variables are measured at each assessment, fidelity ratings are averaged for each provider in the time period between two assessments and the time period leading up to the first measurement. Averaging fidelity ratings between two assessments, the resulting value is treated as a measurement occasion for the later time period (e.g., averaging fidelity ratings for a provider between the first and second assessment is treated as a value for the response at the second assessment).

Sustainment climate response is continuous and, assuming it is normally distributed, will be modeled with an identity link function. The same demographic predictors described above will be modeled, as well as work attitudes, transformational leadership, and organizational climate. Given that responses and predictors are measured at each assessment period, we will lag the response, controlling for baseline measurements [97].

#### Overall mixed-methods integration

We will integrate qualitative/quantitative results through an inclusive process that values the perspectives of all stakeholders (state/county and organizational participants, investigators, consultants). We will assess consistencies and discrepancies between qualitative and quantitative data and analyses to determine if we are capturing issues and constructs most relevant to SC sustainment in the service systems and organizations. We will consider each analysis (qualitative and quantitative) on its own terms and how the two differ or converge in their findings when working towards overall interpretations and conclusions [77],[99],[100]. Qualitative and quantitative data will be integrated through triangulation to examine convergence, expansion, and complementarity of the two data sets [70],[99],[101],[102]. Data sets will be merged by a) linking qualitative and quantitative databases and b) embedding one within the other so that each plays a supportive role for the other. Specifically, results of each data set will be placed side-by-side to examine 1) *convergence* (do results provide the same answer to the same question, e.g., do interview data concur with quantitative data regarding sustainment climate and SC use?), 2) *expansion* (are unanticipated findings produced by one data set explained by another, e.g., can survey data that suggest poor sustainment climate be explained by qualitative interview data?), and 3) *complementarity* (does embedding results of the qualitative analysis within the quantitative data set help contextualize overall results, i.e., does it explain variability represented by confidence intervals or variance estimates in statistical analyses on sustainment climate and leadership?).

#### Aim 4

Integrate findings from Aims 1, 2, and 3 through a collaborative process involving the investigative team, consultants, and system and community-based organization stakeholders in order to further develop and refine a conceptual model of sustainment to guide future research and to provide a resource for service systems to prepare for sustainment as the ultimate goal of the implementation process.

## Discussion

The study of sustainment is at least as important as the study of implementation for a number of reasons. First, the potential public health impact of implementing an EBI will not be fully realized unless that practice can be sustained in the context in which it was implemented. Second, further research is needed (beyond the scope of the present study) to examine initial investments in EBI implementation that may be diminished or wasted without sustainment, resulting in a lack of return on investment (ROI) and a failure to realize cost-effectiveness of EBIs found in other studies. Indeed, while methods for assessing ROI have a long history and despite its importance when considering the sunk costs of implementation efforts [103], ROI is rarely examined. Third, without ongoing practice, skills developed by clinicians will likely be lost or EBI fidelity will be jeopardized [104]. Although flexibility within fidelity is a recommended approach [105], without ongoing feedback or coaching, clinician or service provider behavior may drift from expected standards of practice associated with positive patient or client outcomes. Taken together, such negative impacts of failure to sustain EBIs seriously threaten the value of implementation efforts.

To understand multiple factors that affect implementation and sustainment, mixed methods are needed [101]. For example, gaining a full understanding of the outer context impacting sustainment requires not only interviews with key stakeholders, but an analysis of the written and formalized policies that impact how services are funded, supported, and monitored at the system level. Where services are contracted, an evaluation of requests for proposals, contracts, and statements or work is necessary to better understand the legally binding mechanisms that encourage, support, dictate, or disrupt what services will be provided and in what way they will be provided. In the inner context, mixed methods may inform, converge, or expand the understanding of how leadership, group dynamics, and organizational context can impact the quality with which EBIs are sustained [70]. Finally, studies with prospective design characteristics, such as the one described in this protocol, are needed so that sustainment is not assessed at only one point in time, but rather, changes in policy and organizational contexts can be examined over time, for their impact on EBI sustainment processes and outcomes.

## Authors’ original submitted files for images

Below are the links to the authors’ original submitted files for images. Authors’ original file for figure 1
