# Supplementary figures and images for: Mixed-method study of a conceptual model of evidence-based intervention sustainment across multiple public-sector service settings
Source: Implement Sci. 2014 Dec 10;9:183. doi: 10.1186/s13012-014-0183-z (PMC4272775; doi:10.1186/s13012-014-0183-z)

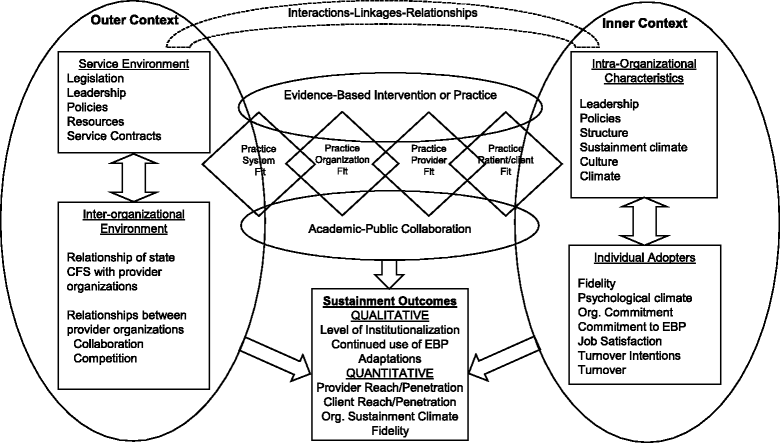

Supplement: Supplementary file 1 — Authors’ original file for figure 1 [file 13012_2014_183_MOESM1_ESM.gif]
